# Editorial: Interactions Promoting Diverse Models of Masculinity and Men's Attractiveness

**DOI:** 10.3389/fpsyg.2021.822921

**Published:** 2022-01-13

**Authors:** Oriol Ríos Gonzalez, Juan Carlos Peña Axt

**Affiliations:** ^1^Departament of Pedagogy, Rovira i Virgili University, Tarragona, Spain; ^2^Facultad de Ciencias Sociales y Humanidades, Universidad Autónoma de Chile, Temuco, Chile

**Keywords:** social interaction, new alternative masculinities, communicative acts, language of desire, gender-based violence

The key role of communicative interaction in the configuration of whom we feel attracted to and the sexual and affective relationships we build, renders it important to analyze in depth which communicative acts promote attraction. The study of the wealth of dialogues, reactions and responses toward these interaction patterns contribute to knowledge and advances in the field of prevention of gender-based violence. Articles selected in this Special Issue address, amongst other topics, the following research questions: How do New Alternative Masculinities (NAM, hereinafter) use language in social interaction regarding sexual-affective relationships? How do NAM men use language in their relationships? Is language use transforming patterns of attraction to violence or care?

A multidisciplinary understanding of how NAM men and the people around them perform communicative acts in relation to sexual attraction emerges as a relevant area of study. This Special Issue contributes to the development of this area with eight articles that examine the essential role of communicative acts produced by NAM men and the impact of such acts in altering expressions of attraction.

Data collection was primarily qualitative and followed the communicative methodology of research, which involves an egalitarian dialogue amongst researchers and social actors (Racionero-Plaza et al., 2021). Whereas, participants contribute their experience to the dialogue, researchers contribute knowledge accumulated by the scientific community on the issue they are addressing. For example, in all of the studies presented in this Special Issue, researchers provided the theoretical basis of masculinity models and attraction patterns, and the participants in the fieldwork applied this knowledge to their own experience. Data analysis not only focused on how communicative acts reproduce the mainstream attraction patterns but also on how these acts can be transformed through the dialogues regarding NAM men. To do that, the communicative research methodology analyzes the exclusionary as well as the transformative components.

The eight articles presented in this Special Issue demonstrate the power of the NAM perspective to subvert the mainstream socialization that leads to dissatisfaction with affective-sexual relationships. Four of the articles analyze the interactions of women who are in fact contributing to reproducing the patriarchal order and the double standard when they blame OTM (Oppressed Traditional Masculinities, hereinafter) men, rather than DTM (Dominant Traditional Masculinities, hereinafter) men, for gender inequalities. Some of the women reprimand OTM men for particular everyday behaviors, treat them as second fiddles and make them men feel insecure with regard to sex. However, NAM men are unmasking the real consequences of such discourse and counteracting them with a powerful language of desire that in fact contributes to overcoming the double standard. Two articles address the positive consequences for the struggle against gender violence of attributing desire to solidary persons. One article emphasizes the need to be consistent in personal commitments to have a truly passionate, egalitarian, and fully satisfying relationship. Another article discusses the usefulness of analyzing popular films to question adolescents' attraction patterns.

The article by Joanpere et al. shows evidence of communicative situations in which NAM men overcome the double standard by rejecting to be with girls who do not “jump for joy” when meeting them. Taking a clear position in favor of egalitarian and passionate relationships and rejecting any affective-sexual interaction that does not fulfill these standards. Analysis of the use of the language of desire and the implications for gender-based violence prevention is also presented.

Schubert et al. explore women's communicative acts that blame all men for what DTM men have done to women and conversely, NAM men's communicative acts that face and stop such blaming. By rejecting guilt and recognizing those men who have always fought on the side of women against violence, NAM men contribute to overcoming hegemonic discourses.

Valls-Carol et al. portrays how NAM men respond to the attacks of women who disdainfully criticize oppressed men's behavior in daily life situations whereas such women would never question this same behavior in dominant traditional men. Again, the use of the language of desire in these responses proves to be useful for eliminating the attractiveness of both DTM men's behavior.

The article by Ruiz-Eugenio et al. shows again how NAM men's position against the double standard confers attractiveness to them. In this case, the situation analyzed is that in which NAM men reject to be the second fiddle to any women.

In “‘No more insecurities’: New Alternative Masculinities' discourse combining desire and equity to tear down offensive sexual statements,” Zubiri-Esnaola et al. analyzes NAM men's communicative acts when they claim what is good and fully satisfying sex: that in which passion, love, desire, and equality are all mixed.

The article by Rodrigues-Mello et al. first addresses the communicative acts that promote attraction to DTM men in the film Three Steps above Heaven, which was a hit with teenagers in Spain. The authors dismantle these communicative acts, demonstrating that all responds to a farce strategy. The article also includes evidence on designed interventions with adolescents in which discussing movies transform perceptions regarding the sexual-affective relationship in the movie.

Pulido et al. demonstrates that, as some men have changed thanks to feminist women, some women have changed their perceptions regarding abusive relationships thanks to NAM men. The results of this analysis note that the use of the language of desire by NAM men with solidary women have become the turning point for women participating in the study.

Duque et al. addresses the crucial importance of the language of desire related to men and women who are successfully helping overcome gender-based violence. Given the close link between attraction and violence in society, the article argues that taking a stance against violence is not sufficient to overcome it. In this respect, the article presents evidence related to some feminist women and NAM men who successfully broke the silence using both types of language.

## Author Contributions

OR has elaborated the introduction of the editorial. JP has prepared a brief description of the eight articles of the Research Topic. Both authors contributed to the article and approved the submitted version.

## Conflict of Interest

The authors declare that the research was conducted in the absence of any commercial or financial relationships that could be construed as a potential conflict of interest.

## Publisher's Note

All claims expressed in this article are solely those of the authors and do not necessarily represent those of their affiliated organizations, or those of the publisher, the editors and the reviewers. Any product that may be evaluated in this article, or claim that may be made by its manufacturer, is not guaranteed or endorsed by the publisher.
